# (Im)mobility and performance of emotions: Chinese international students’ difficult journeys to home during the COVID-19 pandemic

**DOI:** 10.1177/20501579221119585

**Published:** 2022-10-06

**Authors:** Guanqin He, Yijia Zhang

**Affiliations:** Graduate Gender Programme, Department of Media and Culture Studies, 8125 Utrecht University, The Netherlands; Department of Sociology, 8166 University of British Columbia, Canada

**Keywords:** Mobile media, emotion, (im)mobility, international student, Douyin, COVID-19

## Abstract

This article examines mediated performances of emotions by Chinese international students in their transnational journeys returning to China during the COVID-19 pandemic with a focus on the role of mobile media in helping students cope with their cross-border (im)mobility and symbolic immobility. By thematically analyzing 36 self-representational videos produced by returning Chinese students on a burgeoning mobile media platform Douyin, we identify 5 overarching themes of emotional performance: fear, pride, gratitude, shame, and solidarity. We propose that mobile media has the potential to create a hybrid space that witnesses and elicits empathy for the hardship experienced by marginalized mobile groups during the global pandemic. Mobile media, by enabling simultaneous communication, amplifies the sensation of belonging in times of isolation and ambiguity and offers dialogic venues for disparate groups across geographical and socioemotional distances. Our findings suggest the vulnerability of mobile communities in the event of a global pandemic, and the affordances of mobile media in confronting and resolving such precarity. We call attention to the intersections of mobile communities and mobile media amid the global pandemic, particularlyon the experiences and performances of emotions in hybrid spaces.

## Introduction

The COVID-19 pandemic, which caused regional lockdowns across the globe, has greatly impacted everyday mobility around the world. Among the mobile groups depending on transnational mobility infrastructures, international students, who were described as possessing “unlimited global mobility” (Gomes, 2015, p. 46), have experienced a variety of immobilities in the continuing global pandemic. International students are forced to remain in host countries when transnational cross-border mobility infrastructures, such as transportation, regulatory frameworks, institutional coordination, and commercial intermediaries, come to a halt (Chakraborty & Maity, 2020). Stranded in the COVID-19 pandemic, international students experience the unprecedented challenges of remote learning, homelessness, financial hardship (Gallagher et al., 2020), discrimination and racism (Ma Y & Zhan, 2022), and lack of or contradictory information, while suffering from negative emotions such as loneliness, anxiety, frustration, and isolation (Chen J et al., 2020). In this article, we focus on Chinese international students who managed to return to China during the global pandemic. Once valorized as a privileged class with significant geographical and social mobility, these students have become a minoritized group, in both their hostlands and homeland, trapped in transnational (im)mobility (Hu et al., 2022) and symbolic immobility (Smets, 2019).

To begin with, these students experience immense anxiety over the coronavirus. International students, like everyone else, struggle with the virus (Ma H & Miller, 2021). But it is important to recognize that the channels where students obtain information about the fatality, transmission, and prevention of the virus might be different or diversified. Research has found that Chinese international students adopt a polymedia approach and stay connected with families, friends, and both homeland and hostland news through Chinese social media (Yang, 2022). As China was one of the first epicenters of COVID-19, with Wuhan witnessing an unimaginable loss, Chinese students were panicked by the herd immunity policy (Hu et al., 2022) and the less rigorous preventative measures adopted in their host countries, especially compared to the “cruel but effective” countermeasures in China (Graham-Harrison & Guo, 2020). 

In addition to living through the pandemic in a foreign country where the confirmed cases of coronavirus spiked while anti-mask rallies gathered publicly to protest the enforcement of preventative measures, Chinese international students have suffered from high rates of racist attacks since the outbreak of COVID-19 that further exclude them from the host society (Ma Y & Zhan, 2022; Wu et al., 2021). Coronavirus outbreaks in popular destination countries for international students, such as the United States, the United Kingdom, and Australia, have witnessed surging cases of anti-Asian racism and Sinophobia (Shi, 2020; Yu, 2020). For instance, according to The Asian Pacific Policy and Planning Council and Chinese for Affirmative Action, more than 2,100 cases of prejudice and racism against Asians were reported in the first four months of the pandemic (Donaghue, 2020), when Chinese international students were already experiencing high levels of anxiety and insecurity.

**Figure 1.**
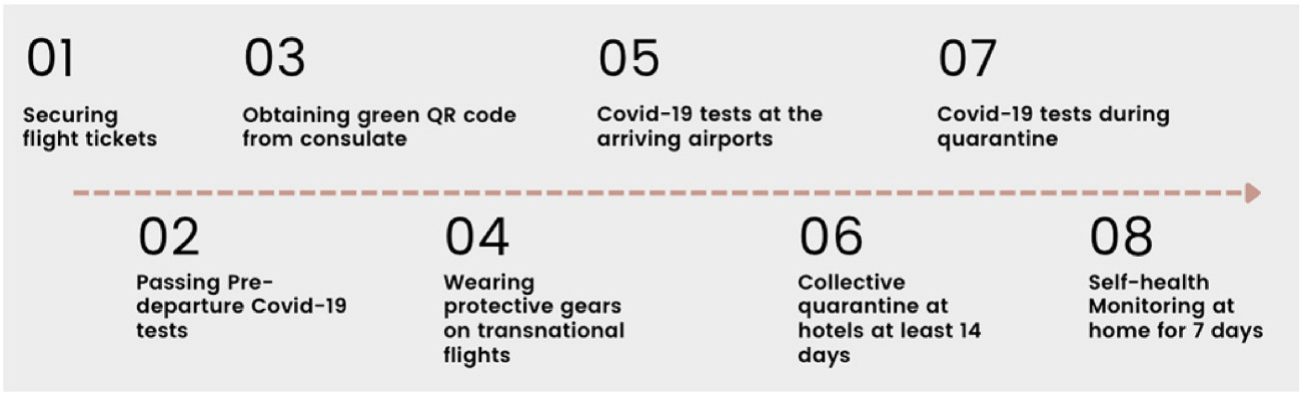
Procedures for overseas students to fly back to China as of June 28, 2022.

Returning home seemed to be a wise choice for many Chinese international students, against the less effective pandemic control and rampant anti-Asian racism in the host countries, especially when they are urged to do so by their families (Hu et al., 2022; Ma H & Miller, 2021). Students, however, face structural barriers obstructing their return journeys and consequently many are stranded in the host countries. To minimize imported cases of COVID-19, the Chinese government encouraged Chinese international students to stay in their host countries and avoid unnecessary international travel (Xu & Zhao, 2021; Zhang, 2021). With international flights severely cut under the “Five One” policy,^1^ students intending to return have to cope with limited flights and expensive airfares. Even worse, their flights may be cancelled at any time. As the number of direct flights decreases, students often have to go through one or more international transfers in their journeys back to China. To board the plane, students need to complete stringent COVID-19 nucleic acid testing (both polymerase chain reaction [PCR] and antibody IgM must be negative) and tedious document preparation. When they finally arrive in China, they have to spend at least 14 days at randomly assigned quarantine hotels (see Figure 1 as of June 28, 2022, summarized by authors). What complicates students’ return journeys is that the policies keep changing and differ from place to place.

In addition to the ruptures of physical mobility, returning Chinese students have to contend with symbolic immobility (Smets, 2019). As indicated in one of the most popular discourses around students’ return journeys during COVID-19, 万里投毒 or “travelling thousands of miles to poison [one’s own motherland]”,^2^ Chinese domestic netizens accuse students of being “irresponsible disseminators of the virus” and “troubles for China’s anti-epidemic workers.” Subject to harsh remarks and hateful speech on the Internet, students are vulnerable to cyber hunting (Cheung, 2009) and consequent cyber violence. In a WeChat report based on stories told by returning Chinese students (Zhou, 2021), students refer to themselves as “Covid-19 refugees”, in light of the months- or years-long ordeal that the group had to endure for their homecoming journeys.

The homecoming of international students has been a heated topic for the general public in China, as well as among Chinese students abroad. Since the first wave of returning students around March 2020, self-representational short-video posts have emerged on the mobile media platform Douyin describing students’ perilous journeys back to their homeland, among other genres of storytelling of the stranded Chinese international students on various platforms (Xu & Zhao, 2021). Mediated by Douyin, students’ physical and symbolic (im)mobility is embodied, performed, and shared to affective communities that may or may not identify with them (Alinejad & Ponzanesi, 2020). Situated at the intersections between mobility and mobile media, these students make and share their experiences on the move, as their mobile emotions transmit, reproduce, and evolve, telling stories of “belonging, identity, and home” (Boccagni & Baldassar, 2015, p. 74). To date, most research on the role of mobile media during COVID-19 has focused on the circulation of information, disinformation, and misinformation online. Studies examining the pandemic-related emotions reported on mobile media tended to concentrate on the immobility associated with lockdowns, social distancing, and mask policies. Our study on the international-student-generated content on Douyin and how students perform their affective returning journeys while navigating divergent expectations for emotional performance adds to this body of literature. In this paper, we introduce emotion as a productive lens to connect migration literature and mobile media scholarship, illustrating how, during the COVID-19 pandemic, Chinese international students use Douyin to create an online space where their experiences and emotions can be witnessed and empathized.

As we will argue, through mediating emotions, mobile media has the potential to create affective publics (Papacharissi, 2015) where the emotional lives of minoritized mobile communities during the pandemic, such as stranded Chinese international students, can be witnessed and empathized. To do so, first, we revisit the migration scholarship and mobile media literature with an emphasis on emotion. Second, we summarize and elaborate five distinct emotional themes accentuated in students’ self-representations of their transnational homecoming journeys during the pandemic. Third, we discuss how mobile media helps students to negotiate with transnational (im)mobility and symbolic immobility, supporting our claim that mobile media can document, express, and transmit emotions, bridging the emotional distances between groups at distinct social situations and transforming the emotional environment where such affective performances take place.

## Theoretical framework

### Emotions on the move

There has been a continuing debate about the nature of emotion, and how it should be distinguished from related terms, in particular, affect (Boccagni & Baldassar, 2015). For scholars like Massumi (2002), affect is automatic and bodily, divorced from the social, while emotion is embedded in social interactions and milieus. Yet recently, interdisciplinary investigations of emotion and affect have witnessed numerous revisits to such distinction. Many scholars discuss emotion as physiological and biological while studying the social causes and consequences of affect. In this paper, we would use affect and emotion interchangeably and follow scholars who define emotion as “not simply ‘within’ or ‘without’” (Ahmed, 2004, p. 25) but as a crucial link between the embodied individual experience and the embedded social participation, connecting the private and the public, the micro and the macro, and agency and structure (Barbalet, 2002; Boccagni & Baldassar, 2015; Turner & Stets, 2005; Zembylas, 2012).

Emotion is an integral aspect of human mobility (Conradson & McKay, 2007; Skrbiš, 2008; Svašek, 2010). On the one hand, as people move from one location to another, emotions become entangled in the process and are constantly negotiated with unfamiliar settings, life circumstances, and points of reference (Boccagni & Baldassar, 2015). On the other hand, “emotions colour, initiate, direct and can stop movement” (Glaveanu & Womersley, 2021, p. 628). Scholars identified a series of emotions associated with different stages of migration. Walsh (2012), for example, associated desire and disappointment with the past, anxiety, and anger in relation to the present, and hope and attachment towards the future. In addition, studies examined nostalgia and longing for the homeland, guilt over the left-behind, and estrangement, alienation, frustration, and regret in the process of adapting to the hostland. Recent scholarship on the affective journeys of refugees zoomed in on the prolonged process in-between the home and the ultimate destination of their migration and pointed out that in this process “hope and despair” are felt simultaneously (Glaveanu & Womersley, 2021). The ambivalence brought by the mix of seemingly contradictory emotions led to refugees feeling stuck, on hold, and in limbo navigating the promise of good life that migratory regimes offered, but inhibited, obstructed, and postponing the means to achieve (Pettit & Ruijtenberg, 2019).

The main body of research on migration and emotion deals with migration as a process that “disassociates individuals from their family and friendship networks, as well as from other socially significant referents that have strong emotional connotations” (Skrbiš, 2008, p. 236). Yet as Skrbiš (2008) points out, in addition to departures and arrivals, “journeys … homecomings and the paradoxes of the migration existence itself” are also key aspects of transnational mobility (p. 241). Chinese international students who managed to return to their motherland during COVID-19, whose digital accounts we study in this paper, serve as a specific case of homecoming, which is an understudied type of affective mobile experience. In this case, students depart from their hostlands where they suffer from exposure to the coronavirus, anti-Asian racism, and everyday stresses of living alone, abroad, and through the pandemic. Yet their destination is not a foreign country where they hope to enjoy better economic prospects or political stability, but their homeland, where rigorous public health measures are forcefully implemented to achieve zero COVID-19 cases and where they are protected from the epidemic, but also stigmatized as selfish overseas nationals who, by returning, bring health risks and socioeconomic burden to the domestic community. Examining the ways students experience, represent, and manage emotions in this reverse direction of migration could contribute to this understudied dimension of affective mobility.

### Emotional turn in mobile media studies

Emotion is a practice. As Scheer (2012) argues, emotions are “a practical engagement with the world” (p. 193). And there are two ways to interpret this practical approach to emotions. One, emotions do things (Ahmed, 2004, p. 26). Two, emotions are a cultural practice—people do, rather than have, emotions (Alinejad & Ponzanesi, 2020; Döveling et al., 2018). On the one hand, being our first point of contact with the world, emotions bind us in relation to space, place, and otherness (Glaveanu & Womersley, 2021). They are our responses to the worlds of others. They align individuals with collectives, depending on the “intensity of their attachments” to their respective communities (Ahmed, 2004, p. 26). On the other hand, emotions are “situational, contextual, and relational” performances in discursively constructed cultural contexts where they form “communities of practice” (Döveling et al., 2018, p. 2). Instead of having emotions, people feel, display, and manage emotions in relation to intersubjective emotion norms (Boccagni & Baldassar, 2015), which distinguish communities from one another (Koschut, 2018).

Defining emotion as a practice productively converges with the affordance approach that conceptualizes media as an “environment of affordances” (Miller & Madianou, 2012, p. 170). The “emotional affordances” of mobile media refers to its capabilities to “enable, prompt and restrict the enactment of particular emotional experiences unfolding in-between the media technology and an actor’s practical sense for its use” (Bareither, 2019, p. 15). The tension between agency and structure makes emotion-as-practice a productive framework when studying emotional mobile lives, which are increasingly penetrated by advanced mobile technologies.

Mobile media and communication enables individuals to weave the flows of connectedness into everyday moments and movements (De Souza e Silva, 2006; Frith, 2015). It makes the networks of belonging portable in the age of transnational mobility (Diminescu, 2008; Leurs & Ponzanesi, 2018). One aspect that stands out is that mobile media facilitates online co-existence (Madianou, 2016; Milne, 2012) and has the potential to build digital interactions between geographically scattered agents. Through digital mediation, geographically distant family members, friends, and even strangers can cohabit at the same time or location, fostering a sense of warmth and belonging (Bonini, 2011) and creating a sense of digital togetherness (Marino, 2015) among those in specific cases of (im)mobility. Witnessing narratives mediated by mobile platforms, for example, are found to help minoritized and stigmatized communities to confront and even transform the physical and symbolic immobility that hampers their everyday life (Witteborn, 2012). The mediated co-presence, however, not only brings the displaced migrants together where they are embraced by an intensified we-feelings (Neag & Supa, 2020). It is equally likely to bring disaligned communities and witness the affective tensions between connected groups and the clash of their respective emotion norms. Özer and Aşcı (2021), for instance, examine how emotions such as fear, anxiety, anger, and contempt are performed in the right-wing populist discourse against refugees circulated on social media.

Mobile media technologies, therefore, offer intricate modes of communicating emotions (Benski & Fisher, 2014), and create new sites where “previously privately shared emotions” are contested and negotiated publicly (Giaxoglou et al., 2017, p. 2). The emotional practices of Chinese international students to document and share their experiences on Douyin and to overcome immobilityto return to their home country could be one of these sites to make interesting observations. The viral algorithm, as a key aspect of the communication infrastructure of Douyin, enables emotional content to travel fast and wide, reaching an audience who may or may not share the authors’ identity. Mediated by such a hybrid, mobile platform that connects heterogeneous communities, how are students’ emotions practiced, performed, and shared? How do students navigate the affective and discursive norms in China that they are stigmatized as burden and threat? What role does the platform play in enabling a co-presence of the minoritized and the normative? Additionally, while studies have documented diverse emotions expressed on mobile media platforms during COVID-19 (Adikari et al., 2021), most of them focused on people’s experiences in lockdowns or the general sentiment change over the course of the global pandemic. Encountering multiple layers of exclusion, mobile populations like migrant workers and international students are incredibly precarious (Kumar et al., 2020; Nanthini, 2020). This article examines the emotional practices of one such group, Chinese international students, who use Douyin, among a myriad of mobile media technologies, to perform and share their affective experiences in the prolonged process of homecoming by overcoming the immobilities brought by COVID-19. By studying their self-representational videos documenting their return journeys, we explore the interplay between mobile media, (im)mobility, and emotions, with a specific emphasis on how mobile media produces witnesses and elicits empathy.

## Methodology

Drawing upon thematic analysis of the short-video posts produced by Chinese international students, this study maps out the themes, issues, and arguments through digitally narrated experiences and discussions regarding their transnational homecoming journeys during the global pandemic. As one of the most popular and successful short-video platforms in China (Chen et al., 2021), Douyin’s daily active users have reached 600 million (Choudhury, 2020). We manually searched for self-representational videos of Chinese international students returning home on Douyin, using the search terms in Chinese: *留学生回国* (International students returning home), *疫情* (COVID-19), and *留学生* (International students). Given the abundance of videos, we obtained data through purposeful sampling (Patton, 2002) on the basis of Douyin’s algorithm recommendation. In total, we gathered 36 self-representational videos of 28 accounts (see Table 1) and the comments these videos received in August 2021. All the names we collected and used throughout the paper are pseudonymized. The sampled short-video posts were uploaded from March 2020 to July 2021, which includes the outbreak of the first wave of COVID-19 worldwide, and the quick spread of the second wave of Delta variants, leading to a massive shutdown of international flights. The short videos dynamically chronicle their transnational journeys, including footages at transient places, pictures of themselves and surroundings, and screenshots of documents and border technology interfaces. To better understand how the COVID-19 situation of the host country is variously implicated in students’ mobile recordings of their desires and strategies of return, we deliberately collected geographically diverse data. In our sample, students’ host countries include, but are not limited to, the United Kingdom, the United States, Malaysia, and Japan.

**Table 1.** List of Douyin short-video posts collected.

Author	Date	Host Country	No. of Videos Collected
Louis	16-Mar-2020	United Kingdom	1
Judy	16-Mar-2020	Spain	1
Qiu	19-Mar-2020 28-Mar-2020	United States	2
Eason	19-Mar-2020	United Kingdom	1
Pengpeng	19-Mar-2020 20-Mar-2020 20-Mar-2020 21-Mar-2020	United Kingdom	4
Vincent	19-Mar-2020	The Netherlands	1
Nannan	20-Mar-2020 29-Mar-2020	United Kingdom	2
Dina	20-Mar-2020	United States	1
Chengzi	20-Mar-2020	Japan	1
Maison	23-Mar-2020 23-Mar-2020 24-Mar-2020	United Kingdom	3
Pili	23-Mar-2020	United States	1
Raven	28-Mar-2020	United States	1
Gina	06-Apr-2020	United States	1
Fei	20-Apr-2020	United States	1
Cici	23-May-2020	United States	1
Landi	20-Jun-2020	United Kingdom	1
Chao	5-Jul-2020	United States	1
Nana	8-Jul-2020	Italy	1
Chuchu	31-Jul-2020	United Kingdom	1
Mandy	30-Sep-2020	United Kingdom	1
Tuni	20-Oct-2020	The Netherlands	1
Yuri	03-Jan-2021	United States	1
Wendy	19-Jan-2021	France	1
Nick	21-Jan-2021	United States	1
Jin	31-Jan-2021	Malaysia	1
Neo	05-Mar-2021 06-Mar-2021	Switzerland	2
Bunny	31-May-2021	Australia	1
Lulu	05-Jun-2021	Singapore	1

Considering the multimedia nature of user-generated content on Douyin, we adopted a comprehensive, intuitive approach to visual data analysis. In addition to the verbal portion of the video, it is equally important to consider non-verbal performances through visuals and background music, among other components (Saldaña, 2013). We first transcribed the videos’ voiceovers and conversations and then coded both verbal and non-verbal elements for thematic analysis. Thematic analysis, as an independent qualitative description technique, is primarily used to identify, analyze, organize, characterize, and report patterns/themes in data corpus (Braun & Clarke, 2006). We follow the six-phase linear process for thematic analysis: familiarizing ourselves with the data; generating initial codes; searching for themes; reviewing themes; defining and naming themes; and producing the report (Braun & Clarke, 2006; Nowell et al., 2017). In the following discussion, we extract five distinct emotional themes from students’ videos, to demonstrate, by sharing their affective journeys on Douyin, how Chinese international students create an online dialogic space where their experiences and feelings can be witnessed and empathized with in response to symbolic (im)mobility during the pandemic.

## Mediated performance of emotions

### Fear

Many students use the hashtag #跑毒 in their video descriptions. Literally translating to “flee from the virus”, this hashtag describes the nature of these international travels succinctly. As journeys to take flight from an unprecedented outbreak of the coronavirus in their host countries, these unusual trips are characterized by several kinds of fears.

**Figure 2.**
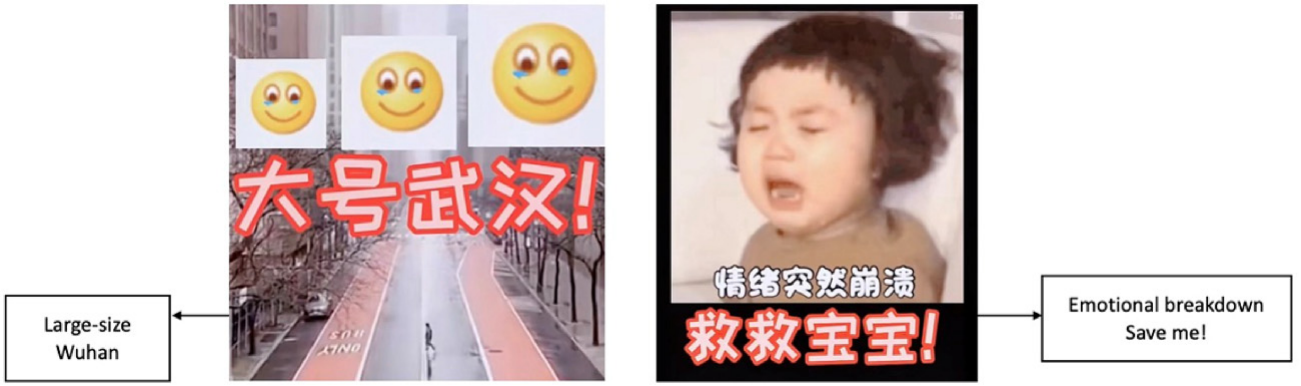
Screenshots of Fei’s Douyin post.

Students are afraid of staying in the host countries. To start with, the local number of confirmed cases is spiking and appalling, especially compared to the number in China. Fei, for example, cited the six-digit cumulative case total for New York State to show her dangerous situation. Filming empty streets, Fei stated in the video: “New York is just a large-sized Wuhan” (see Figure 2). She further expressed her deep despair with an enlarged emoji picturing a little girl helplessly crying (see also Figure 2). In addition, students are scared by the ramifications of local citizens constantly refusing to use protective equipment. A student, for example, cited a local anti-mask rally to suggest the public health hazards brought by such in-person gatherings, which they had to live through as stranded Chinese students.

Fear is also a consistent theme as students prepare for their returning journeys. One of the challenges for students to return is to obtain a flight ticket under the “Five One” policy. Yet even obtaining an overpriced flight ticket does not guarantee a successful return. Many expressed anxieties about their flights getting cancelled even when they arrived at the airports. Naming the trip “an adventure,” Chuchu managed to return eventually after purchasing nine flight tickets via different routes and couriers.

**Figure 3.**
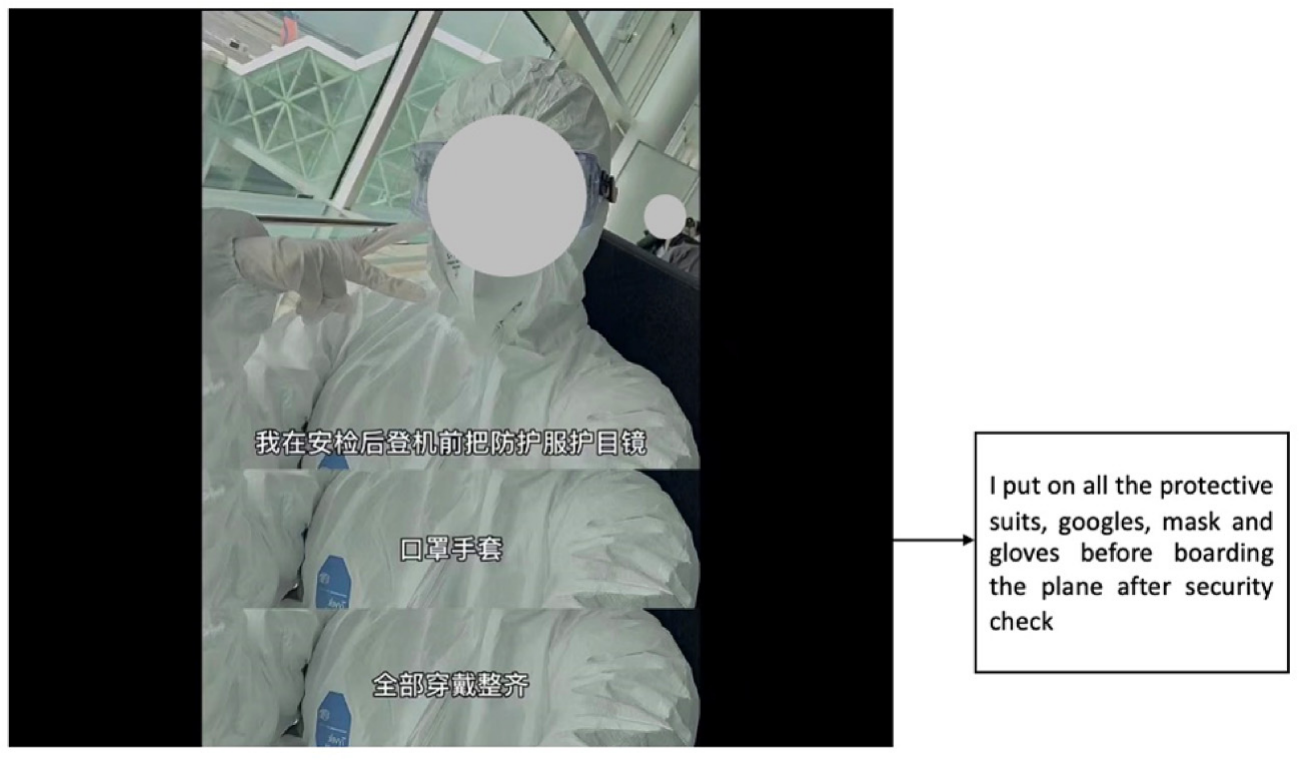
Screenshots of Judy’s Douyin post.

Once the airplane takes off, students can get back to China, which is seen as a safe place where COVID-19 has been under effective control. Fear, nevertheless, still haunts them. Judy, for instance, included a shot of herself clad in a protective suit, mask, and goggles and warned her potential audience that “any inch of your skin should not be exposed in the air” (see Figure 3). In addition to wearing proper protection equipment, Judy suggested that future travellers should refrain from eating, drinking, going to the toilet, or talking during the entire flight. The return journey ranges from a few hours to a few days, and the various kinds of fears experienced and performed by the returning students persist throughout their homecoming journeys.

### Pride

Another key emotion from the videos is pride. Students are proud of the effectiveness of China’s public health efforts against COVID-19, are reassured by the orderly quarantine procedures after arrival, and hold a strong patriotic feeling for their national identity.

Students’ determination to return, to a great extent, builds on their pride in the effectiveness of China’s public health measures against COVID-19, which students believe are not well controlled in their respective host countries. As Mandy commented in her video: “There won’t be any country like China, which is able to systematically fight COVID-19 with the resources of the entire country.”

The sense of security based on students’ national pride emerges as a remarkable emotion in the videos. The returnees expressed their exhilaration upon their return journeys in a myriad of ways. For example, Raven almost “burst into tears” when she realized she was finally sitting on the plane back to China. Landi added smile emoji stickers to pictures of her flight ticket and videos of herself boarding on the plane. Tuni spent one-third of the video on footage of the plane taking off from Amsterdam, accompanied by upbeat music indicating her excitement. Another shared affective tactic to indicate students’ fulfillment of return is the use of the term “finally”—“I am finally on board the plane heading to Beijing”; “I am finally at home”; “[I am] finally back to the arms of the motherland.” “Finally”, despite all uncertainties and immobilities, they were back.

Associated with the excitement of homecoming, Qiu indicated that she felt “a strong sense of safety after getting back to China.” Fei stressed that she was “moved and felt secure” because of China’s preventive measures against COVID-19, following which she expressed her national pride: “A big thanks to the efforts of our motherland. It’s good to be home.” Students are proud of the effective operation of the public health system and the collective efforts of selfless frontline medical workers, whose diligence and dedication were cited by students to explain why they felt secure in China.

A sense of belonging is inextricably intertwined with this sense of security in students' patriotic performances. From the welcome and warmth of the motherland, students found a strong sense of belonging. Louis confessed in his video that he felt at home once the plane landed, as the stewardess greeted him with “Welcome home” in Mandarin the moment he stepped out of the cabin.

**Figure 4.**
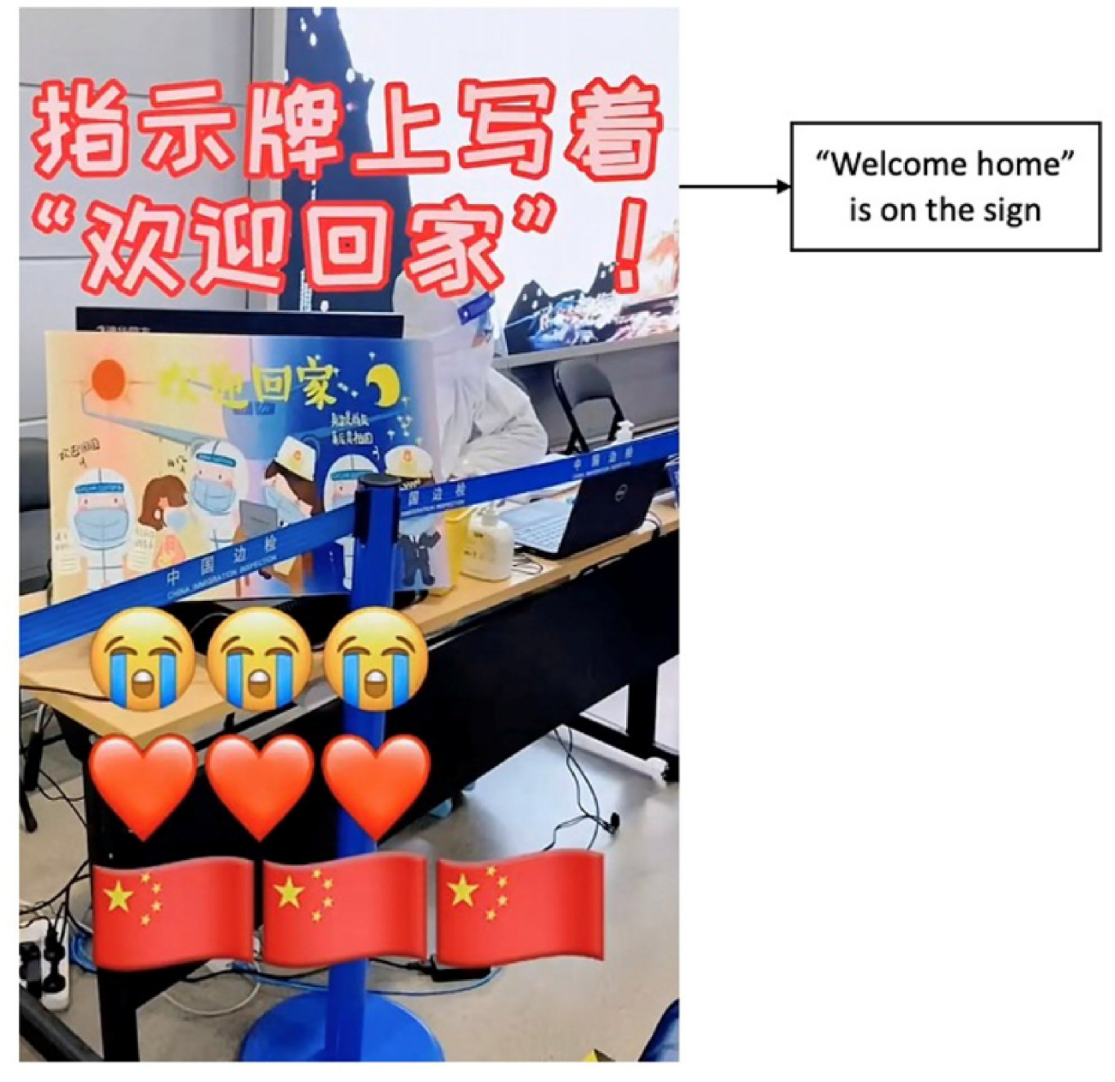
Screenshot of Fei’s Douyin post showing national flag and heart emojis.

In addition to verbal expressions, students used the Chinese flag emojis, grateful signs like heart emojis (see Figure 4), and celebratory music in their patriotic performances. Some used the song “I Love You China” to signal their attachment to their motherland. Two students even used the same climactic line from this song, “I love you China, [my] dear mother.” Students repeatedly referred to China as “motherland” and “home” to whose arms they were eventually returning, and in this way demonstrated their sense of national belonging and patriotic love.

### Gratitude

The third emotional theme is gratitude. Many of the sampled Douyin videos ended with an expression of gratitude to frontline medical workers. Returning students described the frontline workers as patient, diligent, and friendly, emphasizing the selfless care behind their commitment. As Louis interpreted: “Behind the raspy voice was tenderness.” Students showed their appreciation also by suggesting that future travellers should treat these frontline workers with cooperation, respect, and gratitude. Such indebtedness to the frontline efforts is shared among students. Another returning student, Nick, captured a heart-warming scene on the bus transporting returning travellers to their quarantine hotels around 2 a.m. When Nick and his fellow travellers expressed gratitude to a middle-aged frontline worker for her hard work, she stopped, smiled, and said: “No [worries]. You have also worked hard to return home. But seeing you folks appreciate [our hard work], we don’t feel [it is] hard at all.”

Although students may have been aware of the risks that workers confront when receiving them from abroad, the embodied experience of wearing protective gear during the entire flight deepens their compassion for these workers. Pengpeng, for example, spoke to the camera with a selfie shot of himself covered in protective equipment: “Now I just really respect those medical staff working at the frontline in Wuhan who have to wear protective suits every day. It is too difficult. I feel I am about to suffocate.”

This indebtedness to frontline workers is frequently abstracted to, or coincides with, students’ thankfulness to China as their “motherland.” For example, when Louis praised the frontline workers, he added that “[u]nder their protective goggles, there were five-pointed stars in their eyes.” Typically, “five-pointed stars” refers to the stars on the Chinese national flag. Here, by connecting individual workers to a national symbol, Louis depicts frontline workers as representatives of the motherland, taking care of returning students fleeing from zones of epidemic outbreak.

### Shame

**Figure 5.**
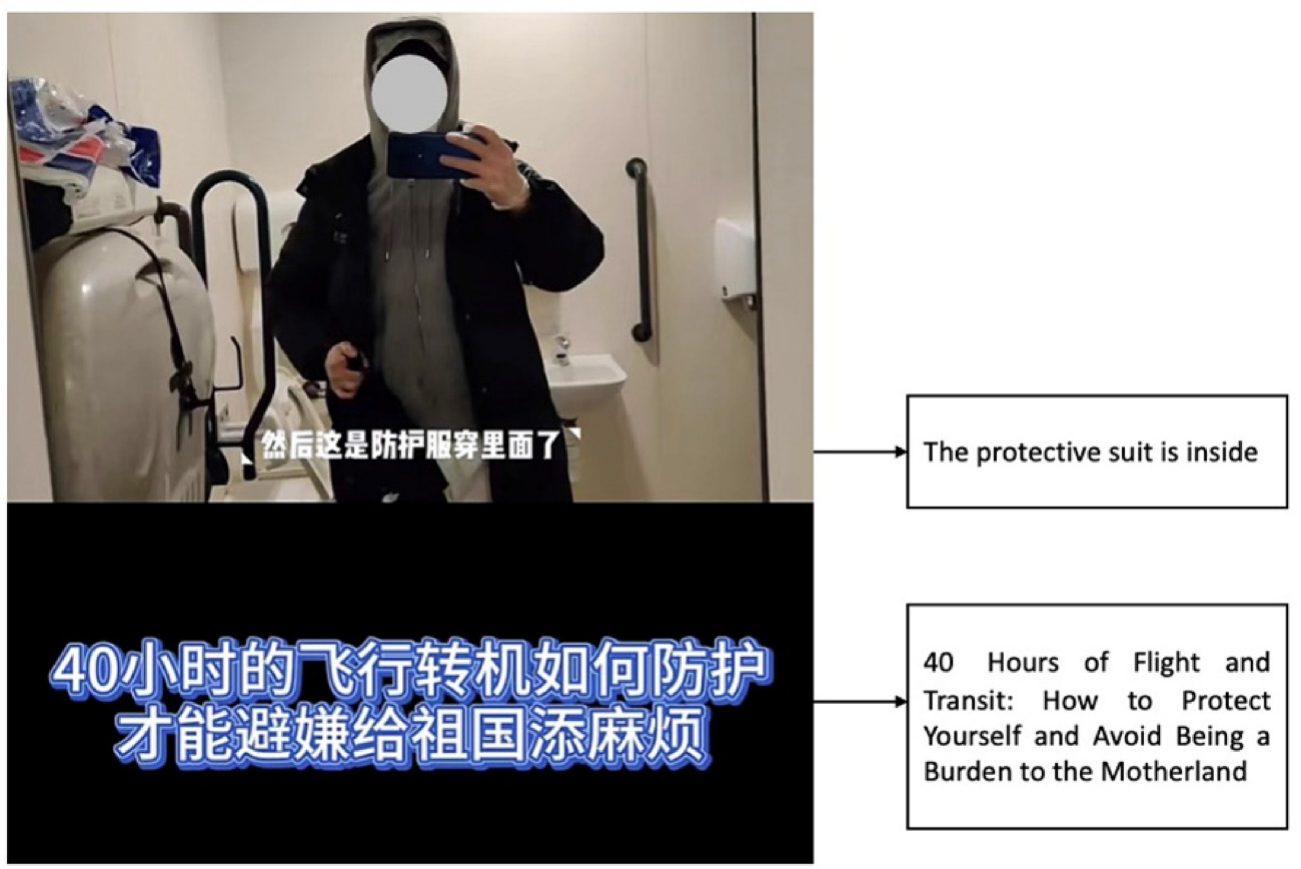
Screenshot of Pengpeng’s Douyin post.

The fourth emotion is shame. As a feeling, it is not as explicit as fear, pride, and gratitude. Nonetheless, it is hard to ignore the guilt and shame returning students bear throughout their homecoming journeys during COVID-19. On the one hand, many international students internalize the stigmatizing discourse. They described themselves as “burdens” to their motherland and their return journeys as causing trouble to the frontline workers. For instance, Pengpeng titled his video “40 Hours of Flight and Transit: How to Protect Yourself and Avoid Being a Burden to the Motherland” (see Figure 5). Echoing this sentiment, Vincent titled his Douyin post, “After A Careful Consideration, I Still Decided to Be a Burden to the Homeland.” He framed his quarantine experience in a positive light, displayed how he led a healthy and productive lifestyle during hotel quarantine by keeping fit and studying, and explained that he spared no effort to avoid adding further trouble for the motherland.

In addition to being performed as internalized guilt, shame is explicitly displayed in the comment section, where returning students are subject to hostile heckling and attacks: “[You] brought back the virus”; “Why did you leave the motherland and now return as if you were fleeing?”; and “Finally we don’t have to wear masks. Why are you coming back?” Such criticism and accusations are liked by many on Douyin, which further stigmatizes the returning students.

### Solidarity

Shame is associated with an affective response of solidarity (Heyd, 2015). People feel solidarity with the communities they align with. Returning students, in general, are united by a shared identity—Chinese international students. While most of them were complete strangers, students supported each other through mobile media platforms like Douyin. Confronting such stigmatization, some commenters stood up for the poster, as well as for each other, attempting to invalidate the stigma. A commenter of Dina, for instance, pointed out that “the worst scenario is that you take all the pains to come back only to find that here you are discriminated against by your compatriots.” In addition to claiming the rights of Chinese nationals abroad to return to their motherland in times of crisis, commenters rebutted the logic of the stigmatizing discourse that returning students are burden to the motherland. As a commenter of Maison contended: “It is reasonable for international students to come home. They are not burdening anyone. They are not supposed to shoulder the country [by not returning]. Raising this issue to a macro level is to put a heavy hat on the student’s head.”

**Figure 6.**
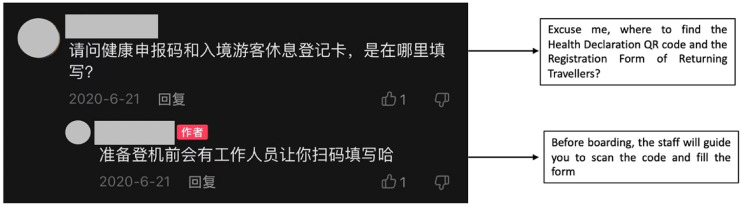
Screenshot of the comment section of Landi’s Douyin post.

Solidarity among returning students is also revealed through information sharing. For example, one commenter of Qiu's post asked if one can pass the security check with a protective suit. Another commenter of Landi's post asked where to find the “Health Declaration QR code” and the “Registration Form of Returning Travellers” mentioned in the video (see Figure 6). It is worth noting that the details and procedures that commenters inquire about are often not readily available from official channels. In turn, the authentic and comparable experiences of the returning students shared in the videos and the comment sections function as a united venue for information exchange and supplementation.

## From witness to empathy

Emotions experienced during or as a result of migration and the associated (im)mobility can be shared (Svašek, 2010). Mobile media, as a communicative structure deeply embedded in the everyday experience, transforms and multiplies the ways in which these emotional mobile lives are shared. On the one hand, mobile technologies produce an “always-on” hybrid reality that redefines the difference between here and there, then and now, online and offline (Boccagni & Baldassar, 2015; De Souza e Silva, 2006), where mobile emotions can be communicated with numerous audiences scattered across different times and heterogeneous locations. On the other hand, given the corporeal nature of emotions, affective performances on mobile media platforms hold a promise to connect unsituated audiences to feel mediated emotions, thereby fostering empathy between previously unaligned social groups. In the case of Chinese international students who return to their homeland during the pandemic, the students rely on Douyin’s affective affordance (Wilkerson et al., 2021), produce accounts of witness to their physical and symbolic vulnerability, and reconstruct the emotional environment of their return journeys through collective efforts. 

### Witnessing

Existing research finds that witnessing narratives produced by vulnerable populations, such as forced migrants in alternative digital spaces may serve to confront and transform the immobility imposed upon them: physical segregation, socio-political exclusion, and discursive stigmatization (Witteborn, 2012). Aptly naming themselves “Covid-19 refugees”, the Chinese international students striving to return to China during the global pandemic experience a similar matrix of immobility brought by their transnational movement, which is perceived as a burden and threat in the populist discourse (Özer & Aşcı, 2021). Building on the existing scholarship on the affordance of digital witnessing accounts of marginalized groups, we outline four layers of witnessing that Douyin, as a mobile media platform, affords in the context of crises and stigmas.

First, students are the most direct witnesses of the immobility Chinese international students suffer in their journeys to return to China during the COVID-19 pandemic, and mobile media crystalizes their witnesses in a digital format that is retrievable and accessible whenever, wherever, and by whomever. Students participate in the entire process of their own returns and have first-hand knowledge of the various “then and there” that constitute the challenging task of return. In addition to experiencing the journeys on their own, students record the various processes of the journeys with footages, pictures, and screenshots, among other media formats. These archived experiences, emotions, and crystalized moments are then assembled, edited, and curated at a slightly retrospective point to produce a Douyin short-video post witnessing what they hashtag as “returning home during Covid-19” and “flee from the virus.”

Second, these individual accounts of immobility connect returning students, who bear a collective witness to their shared experiences. The return videos on Douyin serve as a bridge between students returning to China from different countries, via different airlines, landing at different cities and on different dates. These videos collectively produce a co-existence of students as witnesses of the shared stasis and stigma, enabling the constitution and maintenance of emotional bonds across time and space. The multiplicity of these unique but connected videos amplifies the shared experience and emotions of the returning students.

Third, by watching the mediated witnesses of students, the audience are transformed into co-witnesses of students’ immobility. By co-witness, we mean not only that they can obtain knowledge about the returning students’ difficult journeys shared on Douyin, but that the audience may also see, hear, and feel in the shoes of the students through the embodied performances of fear, pride, gratitude, and shame. In this way, those who did not travel from foreign countries back to China during COVID-19 become witnesses of students’ despair with local public health measures in their host countries, their anxieties about the possibility of flight cancellation, their fears of potential exposure to coronavirus, and their pride in the effective control of COVID-19 in China. These emotions shared by returning students do not contradict the emotion norms inside China at all. Although domestic netizens may rationalize the shame imposed on students, the comment sections of these Douyin videos provide an opportunity for the audience to bear witness to students’ symbolic immobility. Douyin, in this sense, acts as a hybrid emotional environment affording the possibilities of co-witnessing, co-existing, and co-experiencing.

Fourth, as media artifacts recording historical moments (Uricchio, 2009), these Douyin short-video posts, including the comments they have attracted, are witnesses to the vulnerability of groups immobilized during the global pandemic. Epidemic crises are frequently followed by stigmatization and the freezing of mobility of specific groups, some of which are already marginalized (Davtyan et al., 2014). The videos and comments showcase how such immobility unfolds in the life of the immobilized group members and the ways the minoritized react to such immobility. They draw necessary attention to both the students’ stigmatized identity and the discourse that stigmatizes them. These video posts serve as numerous platforms where students and netizens negotiate their misaligned communities of emotional practices, expectations, and aspirations.

### Empathy elicitation

To witness is more than acquiring insider knowledge. Instead, emotion is an essential part of the production and communication of witnessing accounts. In the case of social media-assisted witnesses, emotion plays an even more vital role since social media platforms place a premium on emotion and affect (Wilkerson et al., 2021). Not only do witnesses’ recollections combine with intense affect, but social media users have to curate emotions when producing witnessing content, keeping in mind the audience at whom they are aiming for shares, likes, and comments. Examining the performance of emotions in Chinese students’ return videos, we argue that mobile media platforms like Douyin create opportunities to bridge the emotional distance between the stigmatized community and the broader audience, as well as to foster digital solidarity within the marginalized group.

As discussed earlier, partly owing to the contrast of COVID-19 situations in their home and host countries, Chinese students returning to China experience a change of status from the privileged flexible citizens of high physical and social mobility to a marginalized group whose physical mobility is frozen and whose social identity is stigmatized. In the same process, returning students become the Other in their home country within the populist discourse, where they are the irresponsible virus disseminator and burdens to their motherland. It is within this context that the returning students’ performances of emotions matter, inviting identification from fellow students and broader audiences, as well as undermining the discourse that marginalizes them. Although students’ short-video creations bear witness to their one-of-a-kind experience of coming home during COVID-19, most emotions performed—fear, pride, and gratitude—are not exclusive to returning students’ experiences. Rather, they can all be easily empathized with by the domestic audience. The fear that students experience in face of the spiking number of cases, the slack measures of protection, and the dangers of long-distance flight, resonates with the domestic audience’s anxiety about COVID-19. The pride in China’s counter-coronavirus strategies, the sense of security and belonging upon arrival, and, more broadly, the patriotic love for the motherland echo the collectivist sentiments of the domestic citizens. The gratitude that students express towards the frontline workers is comparable to that of the domestic residents. By resorting to these identifiable feelings, returning students raise the visibility of the emotions that they share with the majority of Chinese netizens, whom they strive to persuade and get included as one of “us”, instead of threatening “them.”

Intertwined but differentiated emotions of shame and solidarity exhibit how these performances of emotions on Douyin assemble and sustain online communities into “networked publics” (Papacharissi, 2015), where the returning students foster a sense of digital togetherness (Marino, 2015) in a way comparable to that of refugees encountering stigmatizing discourses upon their arrival in Europe. An imagined community with a shared identity is constructed (Markham, 2013), giving rise to emotional responses such as friendliness, identification, understanding, and empathy. In contrast to bridging the emotional gap between groups characterized by difference and hostility through appropriation and compromise, the mediated solidarity validates students’ feelings, as well as their desires to return. Performances of solidarity, therefore, offer students a source of strength to confront the hostile environment in which they are situated. Additionally, such solidary performances impress the broader audience with the emotionally charged display of a sense of belonging (Bonini, 2011) among students along with empathic alignment (Döveling & Wasgien, 2015) that may not be prominent in “normal” times but is activated in times of crises such as when students encounter multiple layers of exclusion, minoritization, and immobility.

Mobile media grants greater agency to individual users. Provided with an advanced toolkit of emotional performance, mobile media users like the returning Chinese students are not only creators of their own biographies, as in the case of verbal or textual testimonio (Witteborn, 2012), but curators of the digital display of their affective experience, engaging in dialogue with audiences located at different time-spaces as well as social positions. Emotions are our first point of contact with the world (Glaveanu & Womersley, 2021). Through their synthesis of the body and the place (Bondi et al., 2006), emotions shape the formation of interpersonal and intergroup relationships. By framing their first-hand witnesses with the fear, pride, and gratitude shared by the domestic audience, students bypass the justice debate of their return and resort directly to the intuitive faculties of their audience, decreasing the emotional distance between them and the domestic netizens to such an extent that they may no longer be excluded as threatening “them.” Furthermore, mobile media connects geographically scattered students who otherwise would have been experiencing their immobility solitarily at various corners of the world. And from this togetherness develops solidarity practiced by and empowering the mobile students who are vulnerable to the structural and discursive immobility, as part of the societal reaction to the pandemic.

## Conclusion

The paper explores how Chinese international students cope with their cross-border (im)mobility and symbolic immobility through mediated performances of emotions during COVID-19. Standing at the intersection of emotion, mobile media, and (im)mobility, we zoom in on the most challenging issue confronting Chinese international students during the pandemic: their transnational journeys home. We shed light on how students take the initiative and respond to their pandemic-related (im)mobility, how they document their experiences and emotions through mobile media, and how they construct a dialogic space for their digital solidarity in response to stigmatizing discourse that further marginalizes them.

Building on the scholarship on the digital practices of marginalized groups (Witteborn, 2012), our research demonstrates that affective performances on mobile media raise the voice and visibility of groups minoritized during health and social crises. Enabling constant connectedness across geographical locations (De Souza e Silva, 2006), mobile media energizes a sense of togetherness in times of isolation and ambivalence (Marino, 2015). Its affordances in communicating emotions (Benski & Fisher, 2014; Giaxoglou et al., 2017) and eliciting empathy hold promises to form consensus among groups situated in different social-emotional contexts. Connecting to the literature on migration and emotion, we find the returning students share emotions with precarious migrants taking refuge in a foreign country (Glaveanu & Womersley, 2021; Pettit & Ruijtenberg, 2019), although, compared to refugees, they are moving in an opposite direction to what they call “motherland.” More broadly, we highlight the vulnerability of mobile groups during the worldwide pandemic and the affordances of mobile media to confront and address such vulnerability.

Although we focus on Chinese international students engaging in digital storytelling, connectivity, and solidarity-building within stigmatized contexts, we acknowledge the heterogeneity of the mobile community and especially politics of mobility during COVID-19. The shrinking of the transnational mobility that privileged international students once enjoyed is not comparable to the complete stasis that migrant workers and other historically marginalized groups are trapped in. What we would like to point out is that the mediated performances of returning students on Douyin create opportunities for their experiences to be known, their anxieties to be shared, their indebtedness to be empathized, at a time when domestic institutions and the mainstream discourse aim to contain their mobility—a dire situation faced by most mobile groups including Chinese migrant workers. Taking the Douyin videos posted by returning students as an entry point, we advocate attention to the challenges faced by global mobile communities, their emotional trajectories, and the affordances of bottom-up digital practices.

Last but not least, our analysis is based on self-representational videos produced by Chinese international students who have already managed to return to their homeland. Thus it leaves unexplored students’ motivations, emotional trajectories, and practical challenges hidden behind their creative witnesses. For instance, the returning students internalized themselves as “burdens” in the videos, but we do not know whether they truly consider themselves as burdens or whether this is part of the performative compromise they make in the face of the stigmatizing discourse at home. In this light, in-depth interviews are needed in the future to investigate the individual practices of documenting transnational trips marked by friction and stigma, as well as the heterogeneity within the group of international students along lines of gender, class, and location.

## Notes

1. On March 26, 2020, the Civil Aviation Administration of China implemented a new flight restriction policy enforcing that each (one) country is limited to sending one flight from one carrier over one route once a week, thus “Five One”.

2. All Chinese texts, including phrases, hashtags, captions, and voiceovers, have been translated to English by the authors.

## References

[bibr1-20501579221119585] Adikari A. Nawaratne R. De Silva D. Ranasinghe S. Alahakoon O. Alahakoon D. (2021). Emotions of COVID-19: Content analysis of self-reported information using artificial intelligence. Journal of Medical Internet Research, 23(4), e27341. 10.2196/27341 33819167 PMC8092030

[bibr2-20501579221119585] Ahmed S. (2004). Collective feelings: Or, the impressions left by others. Theory, Culture & Society, 21(2), 25–42. 10.1177/0263276404042133

[bibr3-20501579221119585] Alinejad D. Ponzanesi S. (2020). Migrancy and digital mediations of emotion. International Journal of Cultural Studies, 23(5), 621–638. 10.1177/1367877920933649

[bibr4-20501579221119585] Barbalet J. (Ed.). (2002). Emotions and sociology. Blackwell.

[bibr5-20501579221119585] Bareither C. (2019). Doing emotion through digital media: An ethnographic perspective on media practices and emotional affordances. Ethnologia Europaea, 49(1), 7–23. 10.16995/ee.822

[bibr6-20501579221119585] Benski T. Fisher E. (2014). Introduction: Investigating emotions and the internet. In Benski T. Fisher E. (Eds.), Internet and emotions (pp. 1–14). Routledge.

[bibr7-20501579221119585] Boccagni P. Baldassar L. (2015). Emotions on the move: Mapping the emergent field of emotion and migration. Emotion, Space and Society, 16, 73–80. 10.1016/j.emospa.2015.06.009

[bibr8-20501579221119585] Bondi L. Davidson J. Smith M. (2006). Introduction: Geography’s “emotional turn”. In Davidson J. Bondi L. Smith M. (Eds.), Emotional geographies (pp. 1–16). Ashgate Publishing.

[bibr9-20501579221119585] Bonini T. (2011). The media as “home-making” tools: Life story of a Filipino migrant in Milan. Media, Culture & Society, 33(6), 869–883. 10.1177/0163443711411006

[bibr10-20501579221119585] Braun V. Clarke V. (2006). Using thematic analysis in psychology. Qualitative Research in Psychology, 3(2), 77–101. 10.1191/1478088706qp063oa

[bibr11-20501579221119585] Chakraborty I. Maity P. (2020). COVID-19 outbreak: Migration, effects on society, global environment and prevention. Science of the Total Environment, 728, 138882. 10.1016/j.scitotenv.2020.138882 32335410 PMC7175860

[bibr12-20501579221119585] Chen J. Li Y. Wu A. Tong K. (2020). The overlooked minority: Mental health of international students worldwide under the COVID-19 pandemic and beyond. Asian Journal of Psychiatry, 54, 102333. 10.1016/j.ajp.2020.102333 32795955 PMC7399745

[bibr13-20501579221119585] Chen X. Valdovinos Kaye D. B. Zeng J. (2021). # PositiveEnergy Douyin: Constructing “playful patriotism” in a Chinese short-video application. Chinese Journal of Communication, 14(1), 97–117. 10.1080/17544750.2020.1761848

[bibr14-20501579221119585] Cheung A. (2009). China Internet going wild: Cyber-hunting versus privacy protection. Computer Law and Security Review, 25(3), 275–279. 10.1016/j.clsr.2009.03.007

[bibr15-20501579221119585] Choudhury S. (2020, September 16). The Chinese version of TikTok now has 600 million daily active users. CNBC. https://www.cnbc.com/2020/09/15/bytedance-douyin-has-600-million-daily-active-users.html.

[bibr16-20501579221119585] Conradson D. McKay D. (2007). Translocal subjectivities: Mobility, connection, emotion. Mobilities, 2(2), 167–174. 10.1080/17450100701381524

[bibr17-20501579221119585] Davtyan M. Brown B. Folayan M. (2014). Addressing ebola-related stigma: Lessons learned from HIV/AIDS. Global Health Action, 7(1), 26058. 10.3402/gha.v7.26058 25382685 PMC4225220

[bibr18-20501579221119585] De Souza e Silva A. (2006). From cyber to hybrid: Mobile technologies as interfaces of hybrid spaces. Space and Culture, 9(3), 261–278. 10.1177/1206331206289022

[bibr19-20501579221119585] Diminescu D. (2008). The connected migrant: An epistemological manifesto. Social Science Information, 47(4), 565–579. 10.1177/0539018408096447

[bibr20-20501579221119585] Donaghue E . (2020, July 2). 2,120 hate incidents against Asian Americans reported during coronavirus pandemic. *CBS News. *https://www.cbsnews.com/news/anti-asian-american-hate-incidents-up-racism/

[bibr21-20501579221119585] Döveling K. Harju A. A. Sommer D. (2018). From mediatized emotion to digital affect cultures: New technologies and global flows of emotion. Social Media + Society, 4(1), 1–11. 10.1177/2056305117743141

[bibr22-20501579221119585] Döveling K. Wasgien K. (2015). Suffering in online interactions. In Anderson R. E. (Ed.), World suffering and quality of life (pp. 317–329). Springer.

[bibr23-20501579221119585] Frith J. (2015). Smartphones as locative media. Polity Press.

[bibr24-20501579221119585] Gallagher H. Doherty A. Obonyo M. (2020). International student experiences in Queensland during COVID-19. International Social Work, 63(6), 815–819. 10.1177/0020872820949621

[bibr25-20501579221119585] Giaxoglou K. Döveling K. Pitsillides S. (2017). Networked emotions: Interdisciplinary perspectives on sharing loss online. Journal of Broadcasting & Electronic Media, 61(1), 1–10. 10.1080/08838151.2016.1273927

[bibr26-20501579221119585] Glaveanu V. Womersley G. (2021). Affective mobilities: Migration, emotion and (im)possibility. Mobilities, 16(4), 628–642. 10.1080/17450101.2021.1920337

[bibr27-20501579221119585] Gomes C. (2015). Footloose transients: International students in Australia and their aspirations for transnational mobility after graduation. Crossings: Journal of Migration & Culture, 6(1), 41–57. 10.1386/cjmc.6.1.41_1

[bibr28-20501579221119585] Graham-Harrison E. Guo L. (2020). China coronavirus cases may have been four times official figure, says study. The Guardian. https://www.theguardian.com/world/2020/apr/23/china-coronavirus-cases-might-have-been-four-times-official-figure-says-study.

[bibr29-20501579221119585] Heyd D. (2015). Solidarity: A local, partial and reflective emotion. Diametros, 43, 55–64. 10.13153/diam.43.2015.714

[bibr30-20501579221119585] Hu Y. Xu C. Tu M. (2022). Family-mediated migration infrastructure: Chinese international students and parents navigating (im)mobilities during the COVID-19 pandemic. Chinese Sociological Review, 54(1), 62–87. 10.1080/21620555.2020.1838271

[bibr31-20501579221119585] Koschut S. (2018). Appropriately upset? A methodological framework for tracing the emotion norms of the transatlantic security community. Politics and Governance, 6(4), 125–134. 10.17645/pag.v6i4.1501

[bibr32-20501579221119585] Kumar K. Mehra A. Sahoo S. Nehra R. Grover S. (2020). The psychological impact of COVID-19 pandemic and lockdown on the migrant workers: A cross-sectional survey. Asian Journal of Psychiatry, 53, 102252. 10.1016/j.ajp.2020.102252 32593970 PMC7305726

[bibr33-20501579221119585] Leurs K. Ponzanesi S. (2018). Connected migrants: Encapsulation and cosmopolitanization. Popular Communication, 16(1), 4–20. 10.1080/15405702.2017.1418359

[bibr34-20501579221119585] Ma H. Miller C. (2021). Trapped in a double bind: Chinese overseas student anxiety during the COVID-19 pandemic. Health Communication, 36(13), 1598–1605. 10.1080/10410236.2020.1775439 32530311

[bibr35-20501579221119585] Ma Y. Zhan N. (2022). To mask or not to mask amid the COVID-19 pandemic: How Chinese students in America experience and cope with stigma. Chinese Sociological Review, 54(1), 1–26. 10.1080/21620555.2020.1833712

[bibr36-20501579221119585] Madianou M. (2016). Ambient co-presence: Transnational family practices in polymedia environments. Global Networks, 16(2), 183–201. 10.1111/glob.12105

[bibr37-20501579221119585] Marino S. (2015). Making space, making place: Digital togetherness and the redefinition of migrant identities online. Social Media + Society, 1(2), 2056305115622479. 10.1177/2056305115622479

[bibr38-20501579221119585] Markham A. N. (2013). Remix culture, remix methods: Reframing qualitative inquiry for social media contexts. In Denzin N. Giardina M. (Eds.), Global dimensions of qualitative inquiry (pp. 63–81). Left Coast Press.

[bibr39-20501579221119585] Massumi B. (2002). Parables for the virtual: Movement, affect, sensation. Duke University Press.

[bibr40-20501579221119585] Miller D. Madianou M. (2012). Migration and new media: Transnational families and polymedia. Routledge.

[bibr41-20501579221119585] Milne E. (2012). Letters, postcards, email: Technologies of presence. Routledge.

[bibr42-20501579221119585] Nanthini S. (2020, April 16). Global health security – Impact of COVID-19: Can irregular migrants cope? RSIS Commentary. https://www.rsis.edu.sg/rsis-publication/nts/global-health-security-impact-of-covid-19-can-irregular-migrants-cope/.

[bibr43-20501579221119585] Neag A. Supa M. (2020). Emotional practices of unaccompanied refugee youth on social media. International Journal of Cultural Studies, 23(5), 766–786. 10.1177/1367877920929710

[bibr44-20501579221119585] Nowell L. S. Norris J. M. White D. E. Moules N. J. (2017). Thematic analysis: Striving to meet the trustworthiness criteria. International Journal of Qualitative Methods, 16(1), 1–13. 10.1177/1609406917733847

[bibr45-20501579221119585] Özer Y. Kaçar Aşcı F. (2021). Instrumentalization of emotions and emotion norms: Migration issue in European right-wing populist discourse. Global Affairs, 7(2), 173–192. 10.1080/23340460.2021.1944261

[bibr46-20501579221119585] Papacharissi Z. (2015). Affective publics: Sentiment, technology, and politics. Oxford University Press.

[bibr47-20501579221119585] Patton M. (2002). Qualitative research and evaluation methods. SAGE.

[bibr48-20501579221119585] Pettit H. Ruijtenberg W. (2019). Migration as hope and depression: Existential im/mobilities in and beyond Egypt. Mobilities, 14(5), 730–744. 10.1080/17450101.2019.1609193

[bibr49-20501579221119585] Saldaña J. (2013). The coding manual for qualitative researchers. SAGE.

[bibr50-20501579221119585] Scheer M. (2012). Are emotions a kind of practice (and is that what makes them have a history)? A Bourdieuian approach to understanding emotion. History and Theory, 51(2), 193–220. 10.1111/j.1468-2303.2012.00621.x

[bibr51-20501579221119585] Shi F. D. (2020, October 19). Sinophobia will never be the same after COVID-19. Made In China Journal. https://madeinchinajournal.com/2020/10/19/sinophobia-will-never-be-the-same-after-covid-19/ .

[bibr52-20501579221119585] Skrbiš Z. (2008). Transnational families: Theorising migration, emotions and belonging. Journal of Intercultural Studies, 29(3), 231–246. 10.1080/07256860802169188

[bibr53-20501579221119585] Smets K. (2019). Media and immobility: The affective and symbolic immobility of forced migrants. European Journal of Communication, 34(6), 650–660. 10.1177/0267323119886167

[bibr54-20501579221119585] Svašek M. (2010). On the move: Emotions and human mobility. Journal of Ethnic and Migration Studies, 36(6), 865–880. 10.1080/13691831003643322

[bibr55-20501579221119585] Turner J. Stets J. (2005). Handbook of the sociology of emotions. Cambridge.

[bibr56-20501579221119585] Uricchio W. (2009). Moving beyond the artefact. In Van den Boomen M. Lammes S. Lehmann A. Raessens J. Schafer M. T. (Eds.), Digital material: Tracing new media in everyday life and technology (pp. 135–146). Amsterdam University Press.

[bibr57-20501579221119585] Walsh K. (2012). Emotion and migration: British transnationals in Dubai. Environment and Planning D: Society & Space, 30(1), 43–59. 10.1068/d12409

[bibr58-20501579221119585] Wilkerson H. Riedl M. Whipple K. (2021). Affective affordances: Exploring Facebook reactions as emotional responses to hyperpartisan political news. Digital Journalism, 9(8), 1040–1061. 10.1080/21670811.2021.1899011

[bibr59-20501579221119585] Witteborn S. (2012). Testimonio and spaces of risk: A forced migrant perspective. Cultural Studies, 26(4), 421–441. 10.1080/09502386.2011.587881

[bibr60-20501579221119585] Wu C. Qian Y. Wilkes R. (2021). Anti-Asian discrimination and the Asian-white mental health gap during COVID-19. Ethnic and Racial Studies, 44(5), 819–835. 10.1080/01419870.2020.1851739

[bibr61-20501579221119585] Xu J. Zhao X. (2021). Coping with the “double bind” through vlogging: Pandemic digital citizenship of Chinese international students. Continuum, 36(2), 260–273. 10.1080/10304312.2021.2008319

[bibr62-20501579221119585] Yang F. (2022). From ethnic media to ethno-transnational media: News-focused WeChat subscription accounts in Australia. In Sun W. Yu H. (Eds.), WeChat And the Chinese diaspora: Digital transnationalism in the era of China’s rise (pp. 57–75). Routledge.

[bibr63-20501579221119585] Yu G. (2020, April 10). Racism against Asian mask wearers in rising. It hurts everyone. OZY: A Modern China Company. https://www.ozy.com/news-and-politics/racism-against-asian-mask-wearers-is-rising-too-it-hurts-everyone/301935/.

[bibr64-20501579221119585] Zembylas M. (2012). Transnationalism, migration and emotions: Implications for education. Globalisation, Societies and Education, 10(2), 163–179. 10.1080/14767724.2012.647403

[bibr65-20501579221119585] Zhang Z. (2021). China’s travel restrictions due to COVID-19: An explainer (updated). China Briefing. https://www.china-briefing.com/news/chinas-travel-restrictions-due-to-covid-19-an-explainer/.

[bibr66-20501579221119585] Zhou Y. (2021). 归国留学生的2021: 绿码、熔断与阴阳人 [The year of 2021 for returning international students: Green QR code, flight curtailment, and being tested positive-negative]. The Core Story. https://mp.weixin.qq.com/s/Yu1Co0ogSOzN1Ejj8Ldjew.

